# Prognosis of tetanus patients in the intensive care unit of Provincial Hospital Jason Sendwe, Lubumbashi, DR Congo

**DOI:** 10.11604/pamj.2013.14.93.2180

**Published:** 2013-03-10

**Authors:** Michel Manika Muteya, Alain Kabey a Kabey, Théophile Muyumba Lubanga, Henri Mundongo Tshamba, Albert Mwembo Tambwe a Nkoy

**Affiliations:** 1 Department of anesthesiology-intensive care, Cliniques Universitaires de Lubumbashi, University of Lubumbashi, B P 1825, R.D.Congo; 2 School of Public Health, University of Lubumbashi, BP 1825, DR Congo

**Keywords:** Tetanus, prognosis, mortality, Lubumbashi

## Abstract

Tetanus is still a public health problem in developing countries including the Democratic Republic of Congo. The objective of this study was to determine the prognosis of patients with tetanus admitted in the Intensive Care Unit (ICU) of Provincial Hospital Jason Sendwe, Lubumbashi, DR Congo. This is a descriptive study which collected information from registers and medical records of patients admitted for tetanus in the ICU of Jason Sendwe Hospital, between January 2005 and December 2009. We calculated the parameters of position, dispersion as well as frequencies. We used the test of independent association of prognosis (death versus survival). Tetanus constituted 2.1% of admissions in the ICU during the 5-year period. The average age of patients was 39.38 ± 17; majority of patients were males (95.5%). The majority of patients lived the townships of Kampemba (27.3%), Kenya (22.7%), and Annexe (18.2%). All patients presented the generalized form of the infection. The door of entry was detectable in 71.5% of cases, localized mainly to the lower limbs (61.9%). The average length of stay was 11.29 ± 11.39 days. Mortality was observed in 52.4% of cases. This mortality was statistically significant in patients aged mrore than 40 years (p=0.029) but not not related to the length of stay (p=0.080) nor the location of point of entry(p=0.28). In our environment the prognosis of tetanus remains severe. This disease is still frequent in the city of Lubumbashi; sensibilisation of population on preventive strategies as well as setting up appropriate structures for better management of cases is required.

## Introduction

Tetanus is a toxic infection characterized by a spasm of skeletal muscles that often evolves progressively toward respiratory failure [1]. It is a devastating disease associated with autonomic instability and a high incidence of mortality [2]. Tetanus is caused by *Clostridium tetani*, a bacterium that infects open wounds such as lacerations and penetrating wounds. Older people, women and children are prone to injuries and therefore *Clostridium tetani* at high risk of the disease. The incubation period of the disease varies from 2 to 50 days [2–5]. As there is no specific laboratory test for tetanus, the diagnosis is based on history and clinical signs [6].

WHO and some authors estimate that between five hundred thousand to one million cases of tetanus occur every year worldwide, with 50% to 70% mortality in some regions [3, 7]. The disease has become rare in developed countries mainly due to vaccination programs childhood to adulthood as well as measures of sterilization and aseptic technique [4, 8, 9]. Even in those countries when it is diagnosed, it remains a serious and deadly disease despite the progress of medicine and adequate resuscitation [10].

Although this disease is easily preventable with a highly effective vaccine, tetanus is still common and remains a public health problem; with high mortality in both adults and newborns in almost all developing countries, including the Democratic Republic of Congo [2, 4, 8, 11], years after the introduction of vaccination through the Expanded Program on Immunization.

Lubumbashi is the second politico-administrative city and the more populated city of the Democratic Republic of Congo after Kinshasa. It is divided administratively into seven townships: Annexe, Kamalondo, Kampemba, Katuba, Kenya, Lubumbashi and Ruashi. It counts several health facilities including the Academic Clinics of Lubumbashi, the provincial hospital Janson Sendwe, hospitals of big companies and more referal health centers corresponding to the townships [12].

People affected by tetanus are most often referred to hospital Jason Sendwe because of the availability of anesthetist-resuscitator doctors. The objective of this study was to to determine the prognosis of tetanus in intensive care unit of the provincial hospital Jason Sendwe.

## Methods

### Type and period of study

This is a descriptive study with data coming from the analysis of medical records of patients admitted for tetanus in the intensive care unit of the hospital provincial Jason Sendwe of Lubumbashi over a five years period from January 1^st^ 2005 to December 31^st^ 2009.

### Sampling

It is a exhaustive sampling of all patients admitted for tetanus in ICU. The sample size consisted of 22 patients. The diagnosis of tetanus was based on the presence of at least two signs with or without visible entry door, with or without trismus, sardonic laughing, and rigidity of the abdominal wall and / or neck, in the end by reflex spasm spontaneous or induced by different stimuli. Socio-demographic and clinical parameters such as age, sex, place of residence, the presence of the door, the location of the entry door, the presence of a defect (diabetes, HIV), duration of hospitalization (from day of admission) and outcome (survival or death) were studied.

### Data collection and analysis

Data were collected from registers and patients’ records. Data were encoded using Epi Info ^®^ Version 3.5.1 and Excel 2007 and analysed to derive means, standard deviations and frequencies. Fischer's chi-square was used to assess the association with other outcome variables (age, living, entry door) with a significance level of p < 0.05.

## Results

### Incidence of tetanus

One thousand and twenty-nine (1029) patients were admitted to the ICU of the provincial hospital Jason Sendwe of Lubumbashi between January 2005 and December 2009. Of these, twenty-two (22) cases of tetanus were reported, representing a prevalence of 2.1%. All patients had the generalized form of infection on admission. Medical records could be retrieved for 21 of the 22. The vaccination status of patients was not known.

### Age and sex

The average age of patients was 39, 38 ± 17.79 with a range from 5 to 77 years. 57.2% belong to the 30-50-year age-group; 52.1% of all patients were were younger than 40 years. There were 95.2% of male patients and only 4.7% female (Table 1).

**Table 1:** Distribution of illnesses according to age and the sex

Age	Sex		Total	%
	M	F		
≤20	3	-	3	14.3
21-30	2	-	2	9.5
31-40	6	-	6	28.6
41-50	5	1	6	28.6
≥51	4	-	4	19
Total	20	1	21	100

M= Male ; F= Female

### Municipality of residence

**Figure 1:**
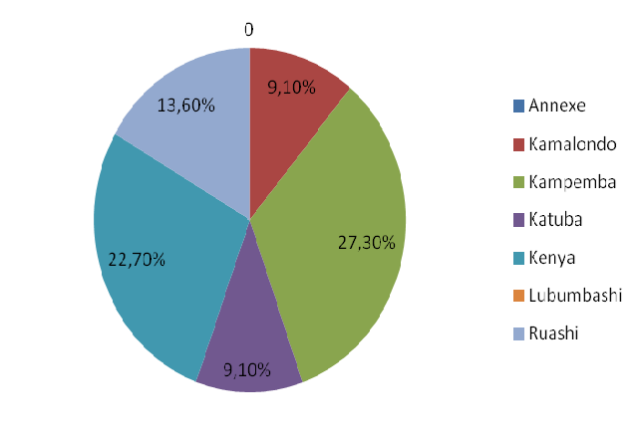
Distribution of the patients according to the township of residence

Figure 1 shows that 27.3% of the patients came from Kampemba town, 22.7% from the municipality of Kenya and 18.2% from Joint Appendix. No patient did come from the center of the city of Lubumbashi.

### Prognostic factors

The majority of patients had detectable point of entry (71.5%) while the point of entry was not detectable in 28.5% of cases. In 61.9% of cases the location of entry was the lower limbs against 4 8% for the upper limbs; in 4.8% of cases, the point of entry was a result of circumcision.

Only one patient was diabetic (4.5%). All patients were treated with diazepam, metronidazole or penicillin and intravenous antitoxin, all this combined with debridement of the wound and general measures of nursing. The average length of hospitalization was 11.29 ± 11.39 days with a range of 1 to 47 days. A mortality of 52.4% was recorded; 47.6% of the patients survived; one patient was discharged from the hospital against medical advice. Mortality was higher in patients aged more than 40 years (P=0.029). The majority of patients who died (81.8%) have a average length of stay of 10 days. Mortality was not influenced by the duration of hospitalization (P=0.80 or the location of the point of entry (Table 2).

**Table 2:** Distribution of patients according to the prognostic factors

	Death	Survival	OR	CI (95%)	P
**Age**					
**<40**	3/21	8/21	0.09	0.01-0.98	0.029
**>40**	8/21	2/21			
**Entry door**					
**UM**	0/15	1/15			0.28
**LM**	9/15	4/15			
**ND**	1/6	5/6			
**Others**	1/15	0/15			
**Stay**					
**<10**	9/21	4/21	6.75	0.96-84.6	0.08
**>10**	2/6				

UM: Upper limbs; LM: Lower limbs; ND: No Determined

## Discussion

Our study focused on tetanus cases admitted in the ICU of the provincial hospital Jason Sendwe, other cases of tetanus treated in other hospital departments including, medicine, surgery, pediatrics and neuropsychiatry were not included.

Tetanus accounted for 2.1% of all admissions to the ICU for a period of 5 years, from January 2005 to December 2009. This proportion is slightly higher than what has been found by Onwudukwa et al, in Nigeria in a university hospital where a prevalence of 1, 36% over a similar period was reported [13]. In reality, this prevalence would be greater if the study was extended throughout the hospital, this was the case in Senegal where, Soumaré et al, found a hospital prevalence of 5.3% [7]. Other cases of tetanus in the city will go unnoticed by because of limited access to health care due to ignorance and poverty of the population. According to WHO, the DRC has an average of 1023.265 tetanus cases reported per year including an average of 764.33 neonatal tetanus per year nationally [14]. This is much higher than in Canada, where 4 cases of tetanus are reported per year, in the United States with 12-15 cases per year, in France with 18 cases per year and in the United Kingdom with 50-60 cases per year [8]. The high prevalence of tetanus in the DRC and in other developing countries can be explained by ignorance, lack of personal hygiene, lack of easy access to primary health care due to poverty and inefficiency of the vaccination program. The majority of patients in this study were male (90.5%) belonging to the age group of 30-50 years. A similar result was reported by Tendesse et al. in Ethiopia [9], Amisi et al. in Kinshasa [12] and Mabula et al. in Tanzania [5]. Simply because they are young adults who are part of the population economically active and and at higher risk of injury. Regarding sex again, the higher proportion of men can be explained by the fact that men are more active handicrafts and exposed to injury and are susceptible to infection, and women are immune due to routine vaccination during pregnancy [4, 15]. Overcrowding, lack of hygiene, ignorance and poor living conditions can explain why the majority of patients are common residents of the largest and most populated and urbanized cities of Lubumbashi: Joint Appendix, Kampemba and Kenya.

On the location of the point of entry, it was detectable in 71.5% of cases and more localized to the lower limbs (61.9%) as is the case in most of the literature on tetanus through the world [4, 9, 12]. This can be explained by the lack of adequate protection (shoes), personal hygiene of patients and once again, ignorance regarding the maintenance of personal health, and poverty.

In our series the mortality was observed in 52, 4% of cases, with a high prevalence in people over 40 years of age. A higher mortality rate was reported by Fawib in Nigeria (57.1%), Ndour et al. in Senegal (60.8%), Mabula et al. in Tanzania (72.7%) and Ilardo et al. in Argentina (75%) [16–19]. By cons, our mortality was relatively higher than what was found in Nigeria, Ethiopia, Senegal and Albania [7, 9, 13, 20, 21]. Decreased immunity from 40 years of age may explain why the disease is more prevalent and the mortality higher in this age group in our study; this finding was corroborated by other authors [12]. High mortality in our study as reported in other similar studies mentioned above, can be explained by the precarious living conditions of the population which does not allow easy access to primary health care, lack of adequate resuscitation equipment (laryngoscope, ventilator, monitor, etc). Beyond all these financial barriers people; the health seeking behavior of the population can contribute to the higher mortality; in most cases, patients will seek treatment from traditional healers before coming to hospital, often late; in hospitals, the limited experience of staff in managinng cases of tetanus can contribute to a higher mortality.

## Conclusion

In our study, tetanus patients represent 2.1% of all admissions ICU patients, predominantly male (95.5%). The mortality rate was 52 4%. The prognosis is generally influenced by the patient's age and the time between injury and medical care. We recommend a vaccination policy from childhood to adulthood by regular booster vaccinations in young adults. Adequate structures, well equipped with resuscitation equipments are needed for better management of patients. Finally awareness campaigns should be conducted for appropriate and timely management of wounds at health facility level, health facility staff should also be capacitated for the appropriate diagnosis and management as well as early transfer to ICU.
